# The Internet of Bodies—alive, connected and collective: the virtual physical future of our bodies and our senses

**DOI:** 10.1007/s00146-020-01137-1

**Published:** 2021-02-08

**Authors:** Ghislaine Boddington

**Affiliations:** Faculty of Liberal Arts and Sciences, Old Royal Naval College, University of Greenwich, Park Row, Greenwich, London, SE10 9LS UK

**Keywords:** Liveness, Telepresence, Virtual bodies, Virtual presence, Personal data, Body data, Biosignals, Biometrics, Digital intimacy, Virtual physical blending, Connected bodies, Identity, Behavioral economics, Prediction economy, Collective, Immersion experiences, The Internet of Bodies

## Abstract

This paper is going to discuss, what will be called, ‘The Internet of Bodies’. Our physical and virtual worlds are blending and shifting our understanding of three key areas: (1) our identities are diversifying, as they become hyper-enhanced and multi-sensory; (2) our collaborations are co-created, immersive and connected; (3) our innovations are diverse and inclusive. It is proposed that our bodies have finally become the interface.

## Introduction

On 16th March 2020, I gave a presentation as part of World Speech Day (2020). It was an entirely online event where speakers from across the globe contributed to several region-specific platforms. They had been asked to share and exchange on the focal topic for 2020: Transformation. World Speech Day has taken this virtual format for a number of years; an event hosted entirely online, with its main goal being to enable access for a diverse global audience. But by the end of the week that followed this year’s World Speech Day, I had agreed to adjust six further presentations planned over the next fortnight from a physical to virtual mode. In the months since, numerous global conferences and symposiums have moved their entire proceedings online and I continue to add 2021 dates to my diary in an ongoing virtual presentation mode.

This shift is all due to the exceptional and complex scenarios created by the continuing COVID-19 pandemic. On an almost unprecedented scale, every human being on the planet has had their life affected by this same significant, universal challenge. To limit its impact on human life, we have all had to instantly change our lives.

Social gatherings, professional meetings, educational lectures and classes, weddings, funerals, doctors’ appointments and birthday parties—planned for many weeks and months ahead—have rapidly moved into the virtual. Suddenly, and pretty much globally, the majority of us must do our work, our learning and our socialising, all from within our homes.

Telepresence—the real-time reception and transmission of audio-visual across time and space—has swiftly consolidated into a mass communication form for use throughout society, and has made virtual presence quotidian. Already available for over the last decade, through apps on our laptops, our mobile phones and smart TVs, video conferencing has been, in 2020, rapidly up-activated to deal with the health requirements needed to stop the spread of this pandemic virus.

In our lifetimes, many of us have watched and sympathised—often from afar—whilst those in distant countries have suffered both human-led and natural disasters. Yet, today, we are faced with an extreme situation of which every human being on the planet has had to take notice, and which has immediate consequences for all of our social, economic and political lives.

The key phrase in use everywhere is Social Distancing. From each other; from other humans and in many countries it has become a legal requisite, in ‘lockdown’. This distancing exists in absolute opposition to our once normal day-to-day human behaviour and more importantly, our innate human disposition.

## The Weave—virtual physical presence design—blending processes for the future

Coming from a performing arts background, dance led, in 1989, I became obsessed with the idea that there must be a way for us to be able to create and collaborate in our groups, across time and space, whenever we were not able to be together physically. The focus of my work, as a director, curator and presenter across the last 30 years, has been on our physical bodies and our data selves and how they have, through the extended use of our bodies into digitally created environments, started to merge and converge, shifting our relationship and understanding of our identity and our selfhood.

Prioritising live presence, yet exploring telepresence and enabling virtual presence through video transmissions, avatars and robotics, I conceived and directed multiple international labs and workshops for performing artists and media artists in the 1990s and early 2000s, exploring together our new representations of self, and examining how we could enable human presence and “liveness” to prevail, above and beyond technological command.

One of the key methodologies that I have been using since the mid-1990s is inter-authored group creation, a process we called The Weave (Boddington 2013a, b). It uses the simple and universal metaphor of braiding, plaiting or weaving three strands of action and intent, these three strands being:

1. The live body—whether that of the performer, the participant, or the public;
2. The technologies of today—our tools of virtually physical reflection;
3. The content—the theme in exploration.

As with a braid or a plait, the three strands must be weaved simultaneously. What is key to this weave is that in any co-creation between the body and technology, the technology cannot work without the body; hence, there will always be virtual/physical blending.

“The imperative is that the creative technical/performance/content is a weave – a three stranded plait that must be kept in continuous and simultaneous motion when in creation to ensure a stable and satisfactory result. This process takes a very different stance to the normal performative creation methodologies. It engages the technical and production participants as artists fully involved in the creative mix. It demands the evolution of a joint pattern of thinking which the whole group needs to form together – a kind of mind-pool of creation patterns which allow the live flow to keep in motion. The results can be highly structured or highly improvised, and the aesthetic diversity that emerges is equally as wide as in other performative forms” Boddington (2000).

My curation of multiple studio-based experiments with dancers and media artists led to numerous remote stage, experimental link ups worldwide from 1995, and gave rise to a series of salient research questions:

- How do projected forms of the body, created and transmitted through digital tools, *change our relationship to ourselves and to others?*
- How does virtual/physical distributed embodiment *redefine identity in socio*-*political terms?*
- How does working and living in virtual space enable and *encourage collective intelligence, collaboration and co*-*creation?*

## Liveness

‘Liveness’ is what we explore in depth as performers; it is not only the presence on the stage, but also the absence. It is held in the memory of performance, within the performers themselves and also within the observers. It is how we create and shift perceptions, as discussed by Peggy Phelan in her book ‘Unmarked the Politics of Performance’ (Phelan 1993).

‘Liveness’ is about intimacy, involvement and interaction. ‘Liveness’ is full of sensory richness; it holds within it a unique real-time connectivity through which we experience each other.

**Fig. 1:**
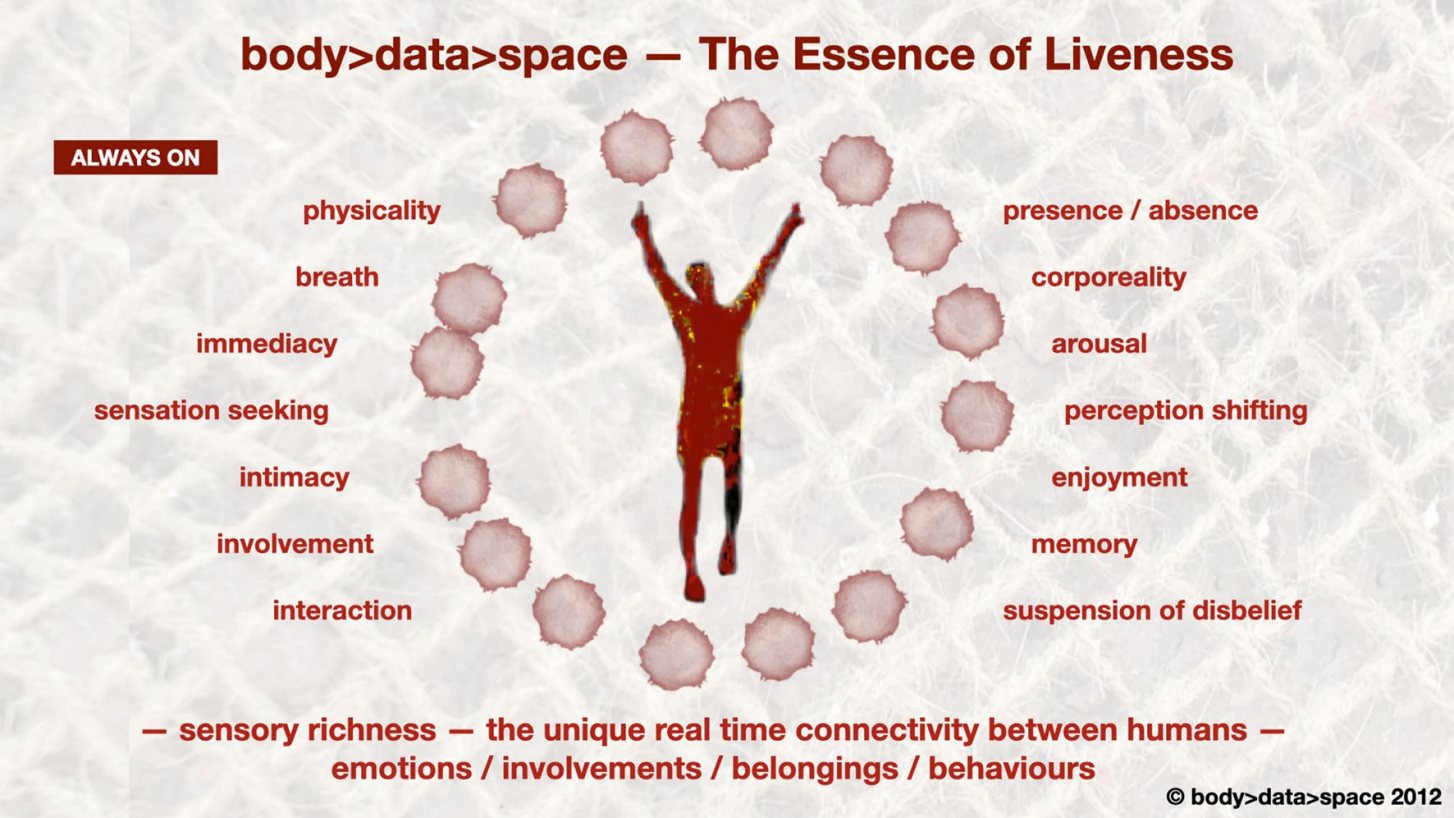
The Essence of Liveness—body > data > space 2012

It ascribes the emotions, the involvement, the belongings and the behaviours that make living beings special; it is the essence of being human (see Fig. 1).

To practice ‘liveness’ requires collaborative networking with others, de-centralised methodologies of working practice that include shared and interdisciplinary ownership. ‘Liveness’ practice uses structured improvisation, it needs a flattening of hierarchies and yet it innately supports specialisation within its collective practice. It attends to virtual and physical applications, is user-generated, and often involves crowdsourcing.

### Hypersensory Self

What we are witnessing today is the emergence of the hypersensory self, blending with the real world. As our senses are digitised and thereby transmittable to others, we expand our sensory capacity through the digital interface we merge with. The body has finally become the interface.

**Fig. 2:**
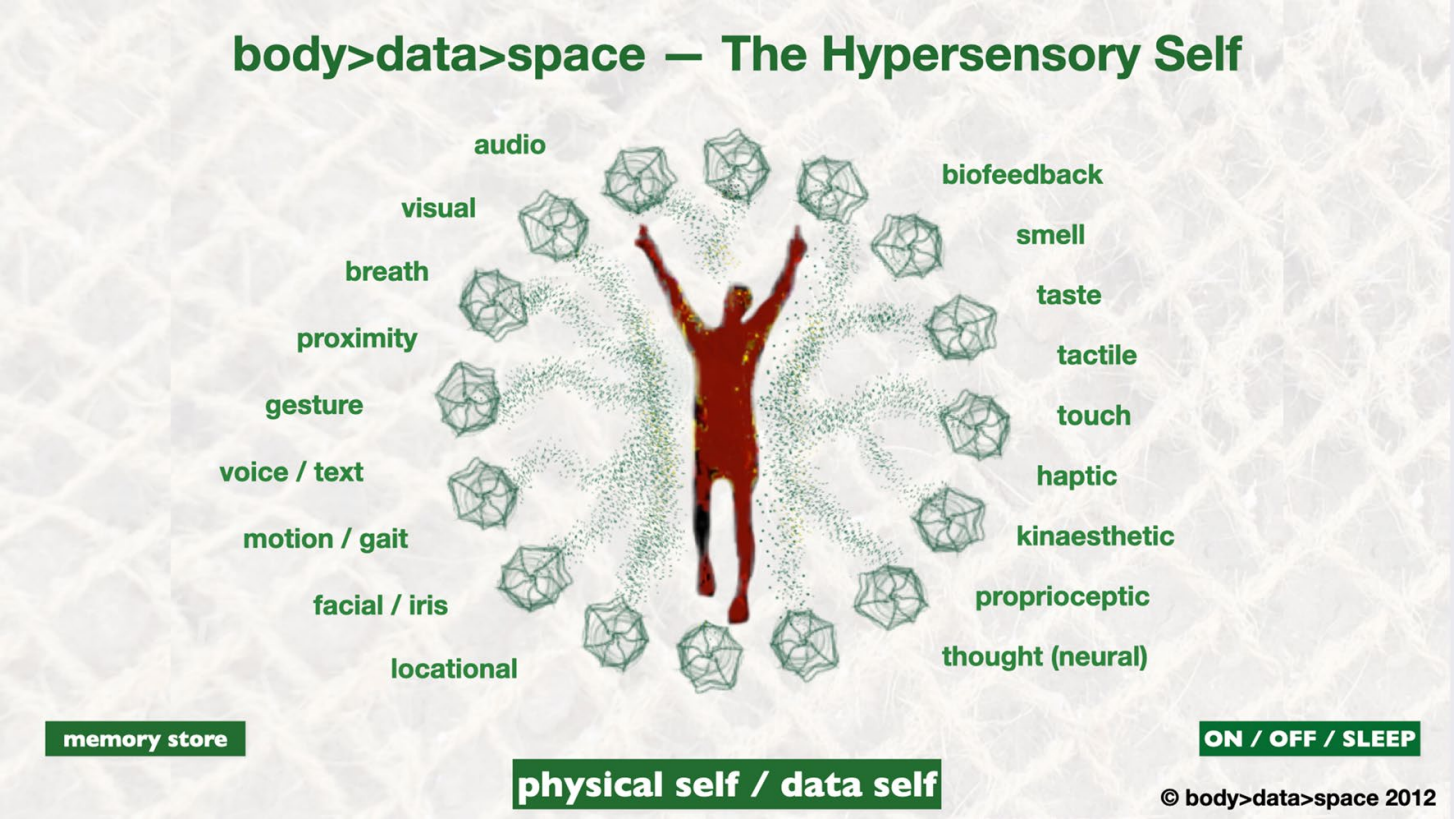
The Hypersensory Self—body > data > space 2012

Figure 2—The Hypersensory Self depicts data being transmitted to and from our bodies. Audio-visual data, data about our location, our proximity and our touch are sent and received via our phones and computers. We use it to our advantage—in its most simple form for two-way interactions. We speak and receive visual data in return, we touch and open up our apps. Facial and iris recognition is at airports to identify us, aiming to replace all passports in the near future. Motion and gait—the way you move, your posture and gesture—is constantly registered by surveillance cameras all around us, examined for ‘unusual’ behaviours, that might suggest we are misbehaving, and to identify  ‘repetitive’ behaviours—harvested by corporations for use in prediction economics.

Many people today are learning to understand, analyse and personally customise these intense data transmissions from their bodies, due to the rapid uptake of biofeedback-led health and well-being wearables and apps, such as FitBit Wearable (2007) and the Apple Health App (2014) mobile phone application. Body activities of all types—walking, cycling, running, heartbeat, respiratory and sleep patterns—are all measured and the data are fed back to us through our devices. People are tracking themselves, some to an extent of an addictive obsession, leading to the emergence of the ‘Quantified Self’, a belief that you can modify or self-experiment with your own body and, through data analysis, change your body into how you wish it to become.

These ‘sensory’ transmissions are often held in our smartphones, and have become highly valuable in our daily lives, for example, real-time location and proximity sensors tell us exactly where we are in the world and guide us accurately as to where we want to go.

The use of touch had, prior to the COVID-19 virus, become extensively used in everyday use of technologies, with one tap access to our phones and apps, as well as touch-led admission to workplaces, lifts and other environments. Touchless technology is now regaining ground, as the virus is believed, to be partly spread through touching the same surfaces as others.

Additionally, we see a rise in the use of gesture and gaze tech, inherent and repetitive body memory actions which start to replace touch and to enable the ‘natural’ usage of the body’s motion to activate data, such as the growth of use of both eyes and arms as controllers within gaming and virtual worlds to determine the direction of travel and for object attraction and repulsion. In this area haptic interactions are all being evolved, at most basic form through vibrations into handheld and other objects.

Smell and taste are in more experimental stages of virtual transmission and reception, with pioneer artists and scientists such as Tillotson (2017) and her eScent wearable device, using biometric stimulus to activate the release of small doses of perfumes to enhance well-being. The Virtual Lemonade experiment was completed in 2017 by a group of researchers “simulated the experience of drinking a glass of lemonade through the digital reconstruction of the beverage’s main visual and taste factors” with the results from these experiments showing the feasibility of “teleporting a glass of lemonade” (Ranasinghe et al. 2017).

Our hyper-sensory self, enhanced by these transmissions and receptions of digital data from within and around the living body, can be seen to be the complimentary extension of the self into this digital era, a “new norm”, implicitly understood by any child born into the developed world since at least the year 2000, with the digital native (born since 1980) pioneering the non-linear, intuitive and participatory learning methods that have been enabled by our new digital tools and networks.

The physical self and the data self are now both fully utilised and engaged together; our body data is being transmitted all around us. We could choose to visualise this as an incredible mass of complex networked patterns, swirling all around us, intersecting and crossing over, looping, overlapping and extending beyond the walls, into the outside space, across cities and countries, mountains and rivers, to reconnect with remote others.

However, it is clear that the difference between our physical and data selves is based in the essence of our liveness, our IRL (in real life) existence as a physical living being. As live beings we are always “on”. Our heartbeats and our breath continue, those vital life signs which are immediately checked in any medical emergency, gives us our deep visceral buzz that is with us continually from birth to death, even when we are asleep.

Yet our technology has an “off” button. In our form as hyper-sensory selves, where we join with the digital and transmit and receive back data, we do have the choice to turn it off. At present, in the evolutionary curve of our merge with the digital, we seem to ‘forget’ we can do this. We are, in 2020, in an intense time of addictive usage of our technologies, extending ourselves into the network, connecting and commenting, requesting and receiving.

Figures 1 and 2 depict the human at the centre of these data interactions, as if we are at the heart of a centralised system built around ourselves.

For the digital technologies sector, the body has not been placed at the centre of attention, as many designers of our daily hardware and software across the last three decades have taken little consideration for the living, breathing, moving body. The lack of expertise and knowledge of embodiment and physical practices, tacit or otherwise, has often been ignored by the design world. They have focussed on the commercialisation of objects and platforms, rather than on the living bodies’ need for complementary tools and enhanced collaborations.

It has been the breakthrough success of mobiles, wearables and implantables that has led the technology industry towards a much needed reflection on the living body and the digital interface. Additionally in 2020, due to the COVID-19 lockdown scenario, we have seen a flourishing of video conferencing platforms to connect us realtime, enabling us to communicate and evolve  the much needed intimacy with each other, rather than with brands and products, that is part of human nature.

### Collective networked collaboration

**Fig. 3:**
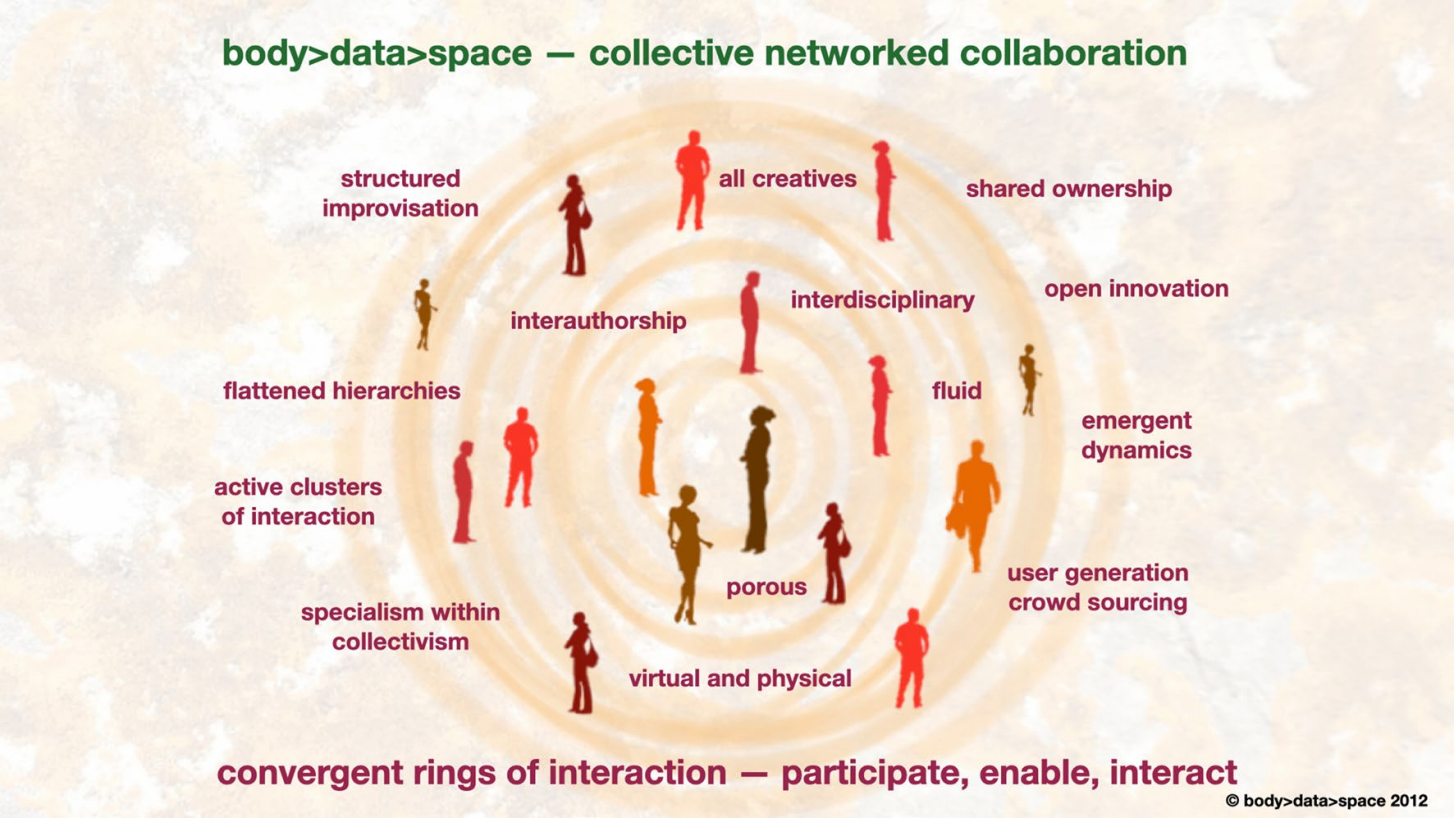
Collective network collaboration—shinkansen/body > data > space 1996

Figure 3—Collective network collaboration illustrates this ‘liveness’ practice in group work. It presents a framework that we apply to either group staged outputs or scaled up for our larger interactive public outputs, such as interactive immersion experiences. Convergent rings of participation here encourage positive interactions by all, a collective networked collaboration between our bodies and through our networked technologies. We utilise our collective networked collaboration methods such as flattening hierarchies while acknowledging specialisation, allowing all participants to have creative input, and enabling, through porosity and emergent dynamics, an iterative yet organic evolution of the process. Structured improvisation techniques, taken from the performing arts, feed deeply into these processes, allowing a highly planned process to still have a creative fluidity at its core, not disabling the innovation that can emerge from diverse groups of people sharing and creating together.

### Human Avatar Cyborg

My fascination from the mid nineties was with how and when we would explore the boundaries of our possible existence through digital extension and enhancement. By the early 2000s, the opportunity to make an avatar of oneself moved beyond the confines of large-scale set-ups for creations of digital bodies used in gaming and animated Hollywood films, into early pioneer virtual worlds such as Second Life (Linden Labs 2003).

Here, I made my first avatar of myself, a huge 8 m giant with purple flowing locks, and had to downsize, with expert in-world instruction, on my first active advances into my parallel universe. Working with choreographers in Second Life to make anti-gravitation dance works took up many hours, but extended my understanding of the deep thread of connection of your living self to your avatar self.

**Fig. 4:**
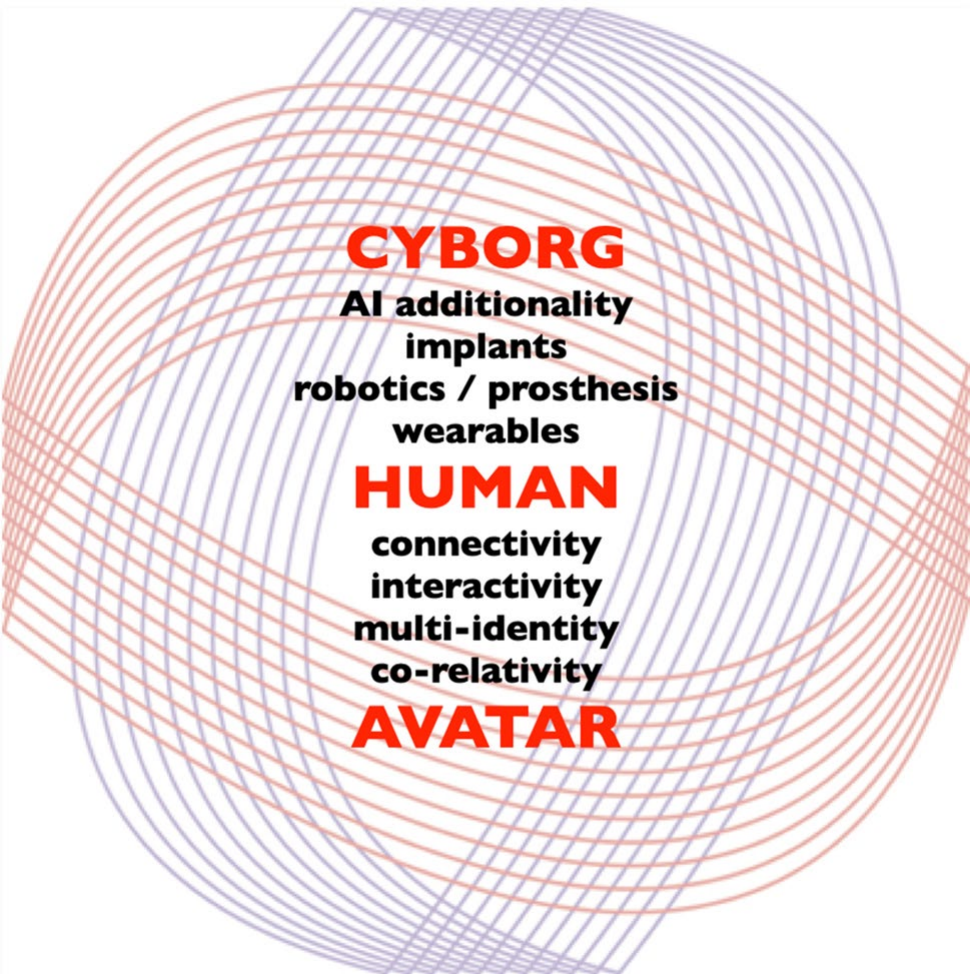
Human Avatar Cyborg—shinkansen/body > data > space 2002

Multiple avatars later I was recognisably beyond the Freudian concept of “oneself” and was happily exploring my multi-self in avatar forms in gaming, virtual worlds and social media environments. This co-relativity, which many of us have had facilitated by and now encounter regularly in our avatar renditions of self, is a difference in psychological engagement with identity; the effect of this on our ability to identify and take responsibility for our actions within the avatar worlds is under debate (Fig. 4).

Today, we head extensively towards the cyborg side of our representation as wearables, robotics, implants and prostheses shift the body into a physical extension and/or merge with our flesh.

## Remote stage connectivity using telepresence: virtual physical blending in the 1990s

In 1991, as part of the shinkansen collective (1989–2004), I started my studio experiments in full body, remote stage telepresence, having drafted my technical plan in 1989 of how to enable this connectivity between studios across space and time. We created connected spaces, initially in the same building, of two dance studios next to each other at Dartington International Summer School in 1991 (Butterfly Effect Network/European Choreographic Forum 1992–1996) using video cameras, screens and BNC (Bayonet Neill–Concelman) cabling—a basic transmission and reception set-up allowing us to see each other real time in each other’s studio and dance together. Across the next 10 years, we did numerous labs and workshops exploring remote stage connectivity with dance, sound and media artists worldwide.

My obsession throughout  the 1990s was that, whilst we learnt to connect, transmit audio and visual in realtime and create complex and improvised choreographed relativity, we still lacked and missed pure visceral performative energy, the essence of liveness (Fig. 1—the essence of liveness). My ultimate aim was ‘virtual physical blending’, a fluidity of identity that would allow us to understand our existence in these parallel worlds simultaneously, and would enable us to evolve a tele-intuitive, intimate stance towards our multi-selves and towards others in the conjoined spaces.

**Fig. 5:**
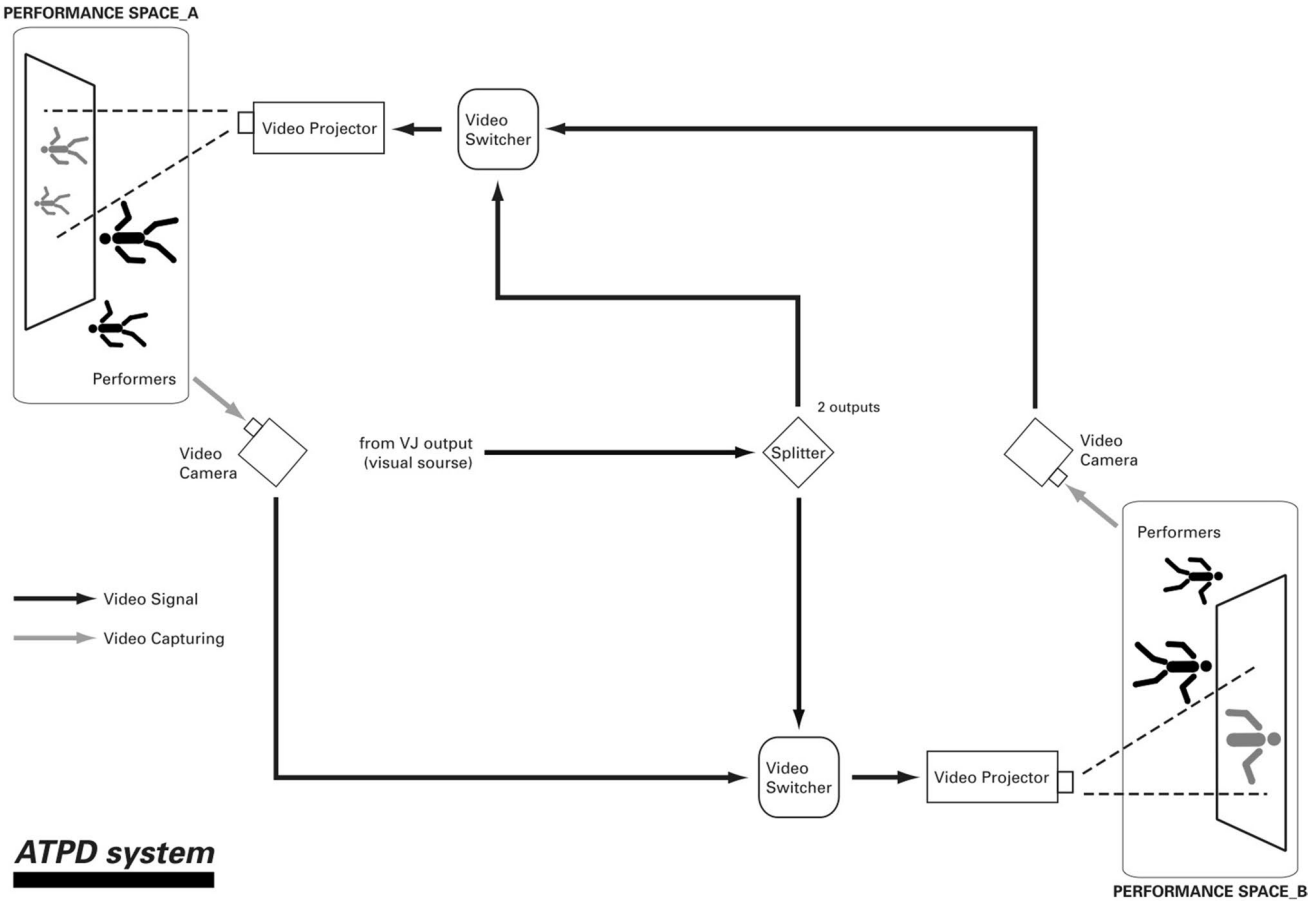
Telepresence—remote stage connectivity—shinkansen/NEST/body > data > space 1997

Expanding techniques learnt from our performing arts training, and working in highly integrated teams of video, sound and movement artists, we wove through the ups and downs, the successes and failures of practice-led research in-studio experiments. As we aimed to convey our presence into the virtual physical space, we felt our way towards a connectivity that was engaging and compelling, a ‘virtual presence’ that enabled us to be together in real time across time and space, and to make our blended virtual physical work (Fig. 5).

### Navigation and orientation of virtual touch

To extend our physical intimacy into virtual forms through telepresence, to build trust and communicate, we tested numerous ways to navigate relativity through screens, to extend beyond our physicality, to send and in return to ‘feel’ the energy coming to us from afar, to create a more tele-intuitive presence, a ‘virtual touch’. Playing with different parts of the body in multiple combinations, we used structured improvisation to shift our normal physical led perception, to find ways to reach an extended embodiment, to exchange and share the experience of existing in both virtual and physical space simultaneously.

**Fig. 6:**
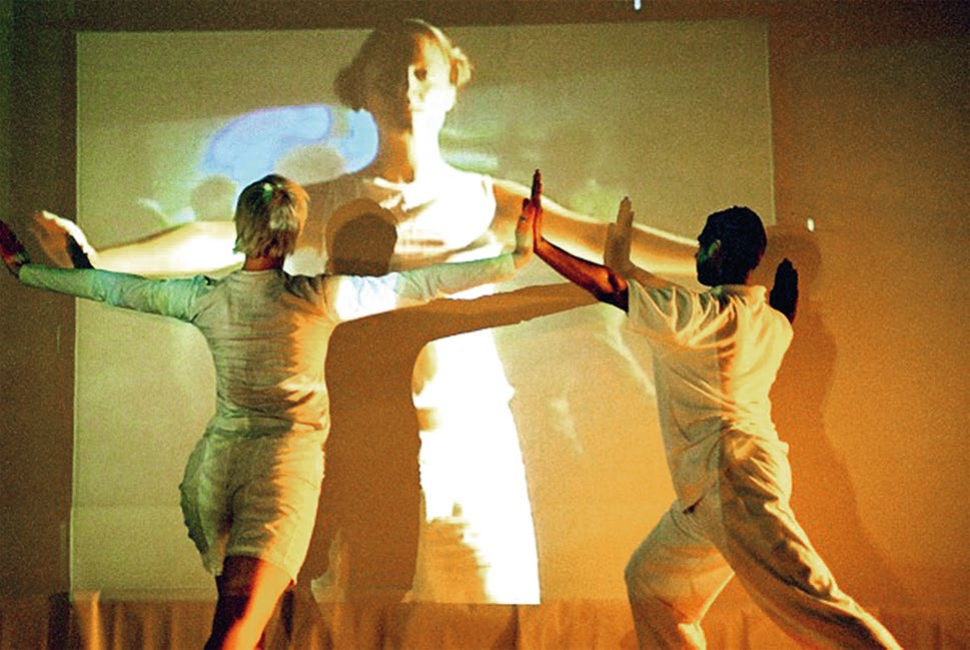
Virtual touch in telepresence—digital intimacy through navigating virtual touch and creating tele-intuition—live connection between London and Bangalore—body > data > space/Akademi 2005

Across the decade, we conducted numerous such experiments. These took days of practice—to line up two remote (non-identical) studios through screens, cameras and the spatial positioning of the dancers, in order to enable this screen based virtual touch, was highly complex and a unique set-up every time. The number of people, skills and machines required at each end of the process made these large group projects requiring interdisciplinary specialists. We learnt and adjusted forward from every lab and every live event, reiterating the work into complex mapping processes of motion, sound and visuals, using detailed timelines held at both ends by the transmission co-ordinators to enable this real-time full-body simultaneous exchange (Fig. 6).

### Lag aesthetics

We were dealing with typical nineties network problems, in majority caused by network latency, which we then called ‘lag’. In the 1990s, lag was normal for audio/visual transmission using the internet, for gaming, for motion graphics in advertisements, for performing arts and consequently the concept of lag aesthetics evolved, used to creative advantage.

We would need to work out the lag at the beginning of each transmission, yet of course it could change regularly, even mid transmission, and when it occurred we learnt that we needed to use it, play with it and make it part of the work. We had to simultaneously count in and out at the end points for every event. For some of our remote stage connected events we would encounter a 0.8 s lag between the distant venues, for the next event it could be a 7 s lag. Luckily musicians and dancers are very able to work with these spatial/time-linked concepts, learning them deep in their bodies memories from years of practice and performance.

### Skin on skin

**Fig. 7:**
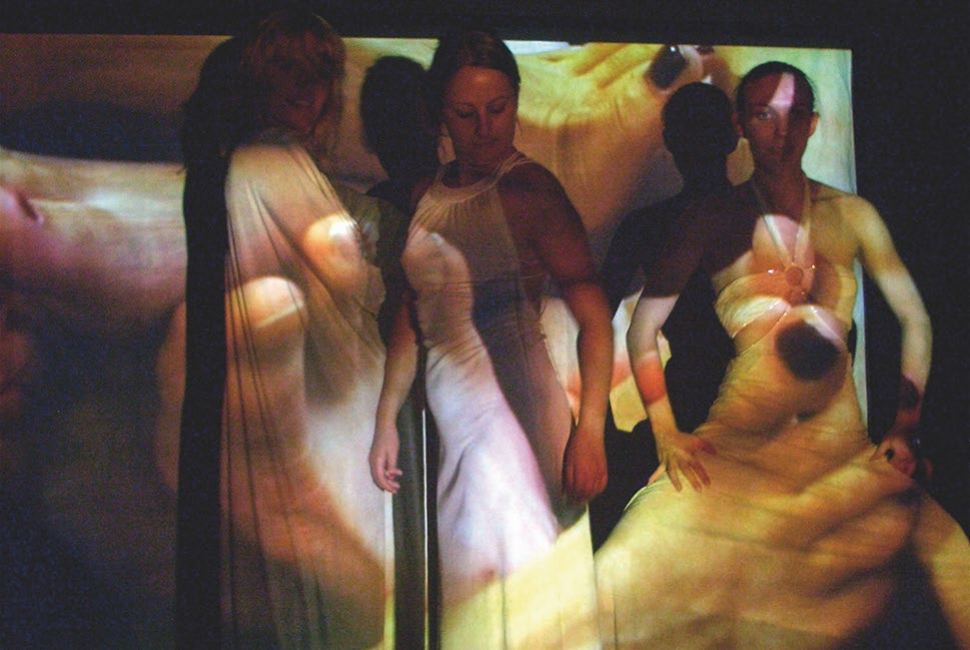
Digital intimacy in telepresence—skintouchfeel—body > data > space collective networked collaboration—the content created was used for a series of outcomes, from performances to installations, virtual and physical 2005–2009

We sought onwards to enable the visceral to be integrated deeply into the transferring imagery, working with the concept of layering skin on skin, creating a virtual cutaneous interaction canvas. By the early 2000s, we were making a variety of works through the compositing of bodies on bodies, trying to reach digital intimacy through creating a repository of gesture-led visual materials called skintouchfeel. We mixed these hundreds of images and tiny videos live into living collages for two way and three-way performances of skintouchfeel, into arts venues, installations and clubs globally (Fig. 7).

“….our skin became our INTERACTION CANVAS, as we connected two, three remote stages for dance performances, exchanging body knowledge, sharing creative ideas and exploring solutions….” Boddington (2013a, b).

The following years expanded into numerous workshops and events using telepresence dance. I convened a symposium in London called Virtual Physical Bodies (1999) which was the first chance to gather international colleagues working with telepresence, alongside our former collective Shinkansen and Wayne McGregor’s Random Dance Company, for a pure knowledge and practice exchange. This led to the CellBytes (2000–2001) series of residencies held between Middlesex University and Arizona State University.

Here at last the infrastructure could support our needs—the university networks were strong and resilient in the main, lag was less of a problem and we were able to stream live not only the outputs from the end points, but a mix of the two/three streams, creating tiny archivable videos of truly integrated virtual physical creations.

## The integration of avatars, robots and sense tech

### Avatars

Our fascination with representation of self and other through digital forms was ongoing and in 2005, we reformed (from the shinkansen collective)  to become the body > data > space collective, intent on delving deeper into the connectivity between our bodies, our data and virtual physical space. We created our own active avatar Orla Ray (2009) and she could dance with us real time on stage. She could change size, relate and activate, and work with us into installations and other participatory immersion experiences. She (still) lives within a hard drive and comes out for different projects. E.g. Post-Me New-ID (2009).

### Robotics

Exploring deeper the representation of our bodies and the hyper-sensory engagement that we believed could be enabled through the digital, we worked with robotics regularly. In one commission, The Blind Robot by artist and robot scientist Demers (2011), the robot had no head; it was constructed as a body and long arms with hands and fingers attached. When you took up the opportunity to sit in front of The Blind Robot it gently touched and explored your face. It had, within its fingers, the ability to extend familiarity by drawing your face on a nearby screen but, in fact, we very quickly found that the public were not interested in face drawing; they were utterly and eerily fascinated by experiencing the ‘touch’ of a robot on their skin. This public touch, robotic installation work travelled around the world to Ars Electronica and into FutureFest London 2015, and then into more commercial scenarios, such as ITU Telecom World 2013.

Making its point about the shift in our engagement in intimate experiences of the ‘other’, The Blind Robot very successfully hit a sweet spot at the time within the moving curve of the “uncanny valley”. “I’m being touched by a robot” was, and still is, an unusual occurrence. We do, in fact, touch or are touched by many robots in our lives, from cashpoint machines to entry sensors, from robotic-led medical interventions to mobility aid devices. Yet The Blind Robot was a humanoid robot with hands and fingers, albeit without a head, and this extended its “reflection” effect. This commission enabled access for the wide audience into a debate normally held in academia alone.

### Gesture tech

My exploration of what I see as the expanding Internet of Bodies has, at its core. the use of multi-modal data feeds from our personal biosignals. Gesture tech is a growing technology sector emerging from gaming and motion capture, and alongside gaze tech,  it is particularly important now in the COVID-19 era, as touchless rules become fixed in order to lessen virus spread.

In 1989, Chouinard (1987), a now internationally acclaimed Canadian choreographer, dances with sensors hidden in a costume that created an extension to her leg calf, with additional sensors placed within her head dress. Her costume activated sound and light on stage through her gestures and movements within the stage space.

**Fig. 8:**
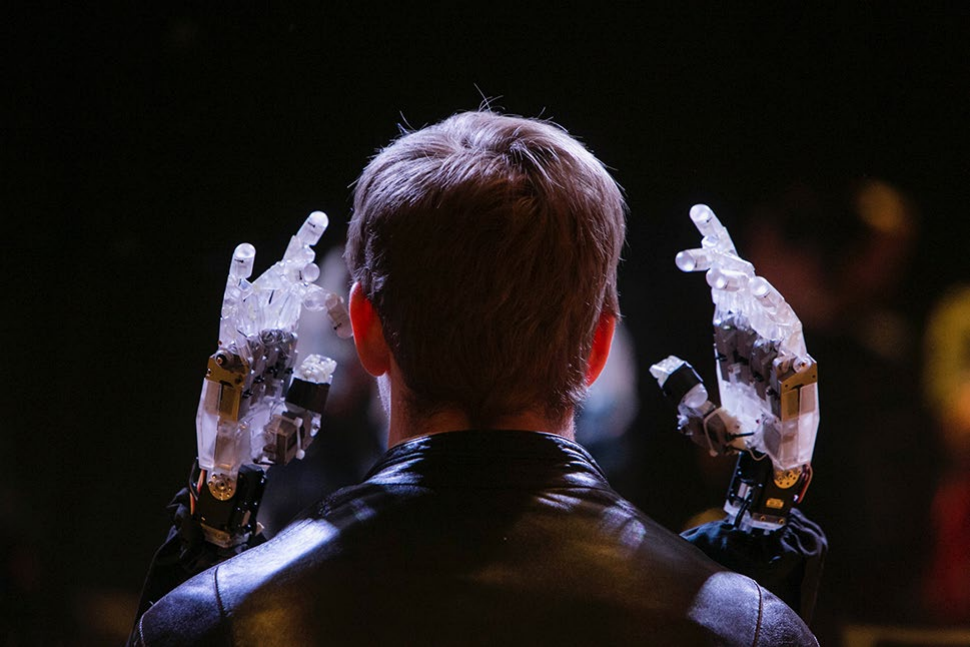
Digital intimacy with robots—The Blind Robot—Louis Philippe Demers—as part of the Robots and Avatars programme for EU Culture programme—body > data > space/KIBLA/AltArt commission 2009

In 2016, Italian artist Marco Donnarumma (2016), who works by converging performance, biotechnologies and computer music, created Corpus Nil, a work focussed on proprioception. In this work a band placed around his arm picks up a range of different bio-signals from his body. He takes the data gathered from his, often minute, muscle movements, from the sound of his blood flow in his body, from his breath and his heartbeat, and he turns this data into performance environments, creating incredibly complex audio-visual experiences to surround and immerse around him and the audience within the event space (Fig. 8).

The artist Trubat (2014) created an innovative app-based concept called E-Traces to turn a pointe ballet shoe into a digital paintbrush. A couple of motion sensors are attached to a ballet shoe and linked to an app, allowing you to view the floor patterns as you dance across studios and stages. Anyone in the dance world will know that this is an incredible step forward, as dancers need to learn to conceptually spatialise in their heads and memorise not only the steps and interactions with others, but the detailed floor patterns. This is one of the most complex body mind interfaces of choreographed dancing, particularly when working in large groups on stage to recreate intricate contemporary performance works. E-Traces is a radical use of motion sensors, enabling the visualisation of the previously “invisible” for reconstruction and rehearsal of dance works.

### Motion capture

In the early 1990s, we could experiment with early motion interaction tools such as full body theremin and lasers/triggers. By the mid to late 1990s motion capture systems had become more widely available, beyond the high-cost set-ups used for use for military, big Hollywood films and for gaming creators such as EA Sports. Hoewever the systems available still ‘tethered’ our bodies to the ground on long wires, or restricted us within huge scaffold cubes of motion capture cameras and lights, disabling the dancer from moving dynamically within or across space, and only enabling outputs as pre-production data, to use post processing as projected imagery into the real-time stage spaces.

Software started to emerge from the early 1990s that enabled choreographers to record, or capture, their own movement data directly allowing, for example, the motion of the fingers moving could  be mapped onto a leg. LifeForms, developed by international media artist Thecla Schiphorst, was used by Merce Cunningham from 1989 and converged with Poser (1995) by the mid 1990s to be utilsed creatively by then upcoming choreographers such as Wayne McGregor for his break through Trilogy of works (1999–2001).

“One CNN reporter introduced a clip on LifeForms by saying, “Finally technology is coming to the rescue of choreographers.” I never imagined technology rescuing choreographers. It’s really the opposite: the nonlinguistic knowledge inherent in physical training is a richly technical world that can inform technological development. One reason LifeForms operates so well is that our bodies work so well.” Thecla Schiphorst, Wired (1996).

The best example at this time of the complexity of the use of real physical motion to processed motion capture and back again (to real-time motion) was the culmination of Merce Cunningham’s work (at 80 years old) called ‘Biped’ (1999). In this work, he used Lifeforms and other software to generate movement ideas, and pre-produced images of the moving dancers to project onto scrims placed in multiple angles across the stage space. He then ‘reinserted’ the live dancers who were choreographed precisely to relate, in real time, to these reflections of themselves floating with and around them on the stage.

However, it was the launch of the Microsoft Kinect system in 2010 that enabled body > data > space, and many others involved in creating interactive real-time experiences, to move into a genuine experiential space, finally allowing the inexpensive use of motion capture in real time.

Microsoft Kinect (2010), a gaming motion capture system created for use as a hands free, wearables free, wires free, user interface for Microsoft’s Xbox, changed at a mass level changed people’s understanding of motion capture—they gained the tacit knowledge that if you did a particular movement, your avatar on screen would do it too, in reflection of you, clarifying the fact that …magically it seemed… your avatar is you!

### Convergence—virtual world, telepresence, motion capture and gesture tech

**Fig. 9:**
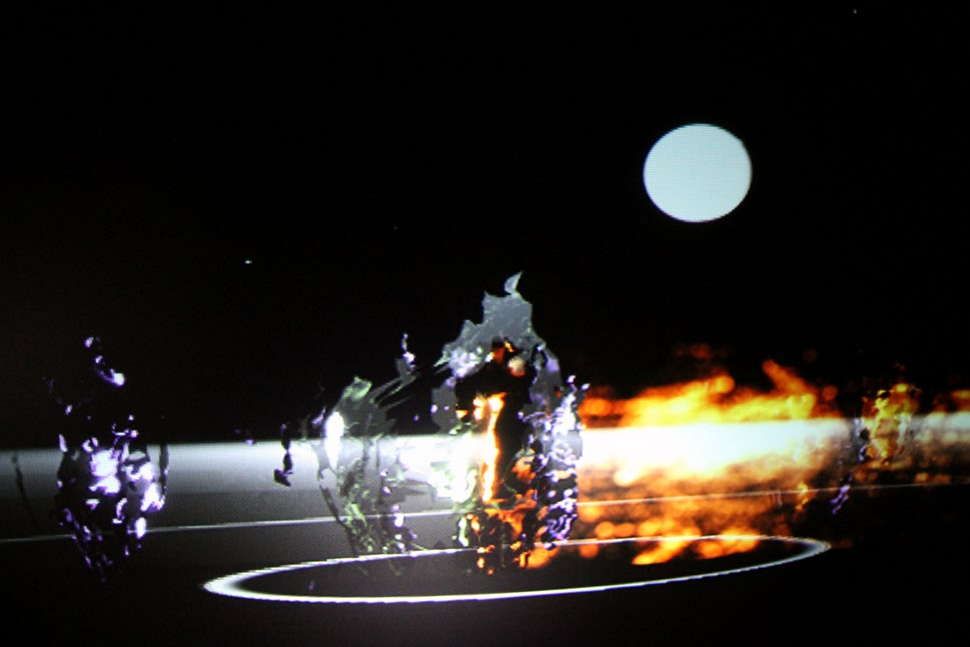
me and my shadow—London, Brussels, Paris, Istanbul as part of the MADE programme for EU Culture programme —body > data > space/National Theatre commission 2009

In 2012, we made me and my Shadow (2012) (see Fig. 9) with the National Theatre in London and European partners. In this large virtual world, we used convergence of human gesture, telepresence, motion capture, avatar creation and surround sound. It was in the connected virtual world that you met with others for full body immersion, as we streamed it live between participants in London, Paris, Istanbul and Brussels.

In this work you enter the black box ‘portal’ and become an avatar in the virtual world with the other three people from the distant cities. Each active avatar was portrayed by a ripple, which showed your ‘liveness’. We gave people a very simple instruction; navigate to the middle of the virtual world, indicated by a large animated full moon,  using simple shoulder and head movements and back/forward steps, and find each other and hug. In reality, as in all our telepresence in the 1990s, you could not actually hug each other; as a virtual avatar you fluidly pass through each other. However the aim for remote intimacy, and the effect of this aim on the over 9000 participants was conceptually mind shifting.

Me and my shadow “points to a much more interesting future where the boundaries between the real and the virtual disappear (…) sometimes you are in the virtual space and sometimes in the real world and you cannot tell the difference” Bill Thompson on BBC World Service, Click 2012.

## The Internet of Bodies—the future of collective co-creation, where the virtual and physical merge

Our work me and my shadow and the feedback we received from this connected installation, a world-first in many ways, enabled a significant move forward in our long term conceptual thinking about virtual physical blended scenarios. Our aim to inter-connect physical bodies was realised through avatar creation of self, enabled to move, within an action-perception feedback loop, with other physical beings/avatars at a distance, all within a real-time avatar environment.

The Internet of Bodies visions an interconnectivity led by our bodies and our identities, where our transmission and reception of data exist within co-created virtual physical space, where our physical selves are ‘tethered’ to our data selves.

Within this space, it is mainly seen as the job of technology to enable the smooth interaction and the inter-connectivity, yet we were confirming to ourselves as creators of interactive works, through numerous public experiments, that the living body was by far the most important element in the mix. Whether using telepresence, mixed reality scenarios, gaming platforms or AR, the focus must be on being inherently and simultaneously alive, connected and collective, when both physically and virtually present.

### Collaborative co-creation space

**Fig. 10:**
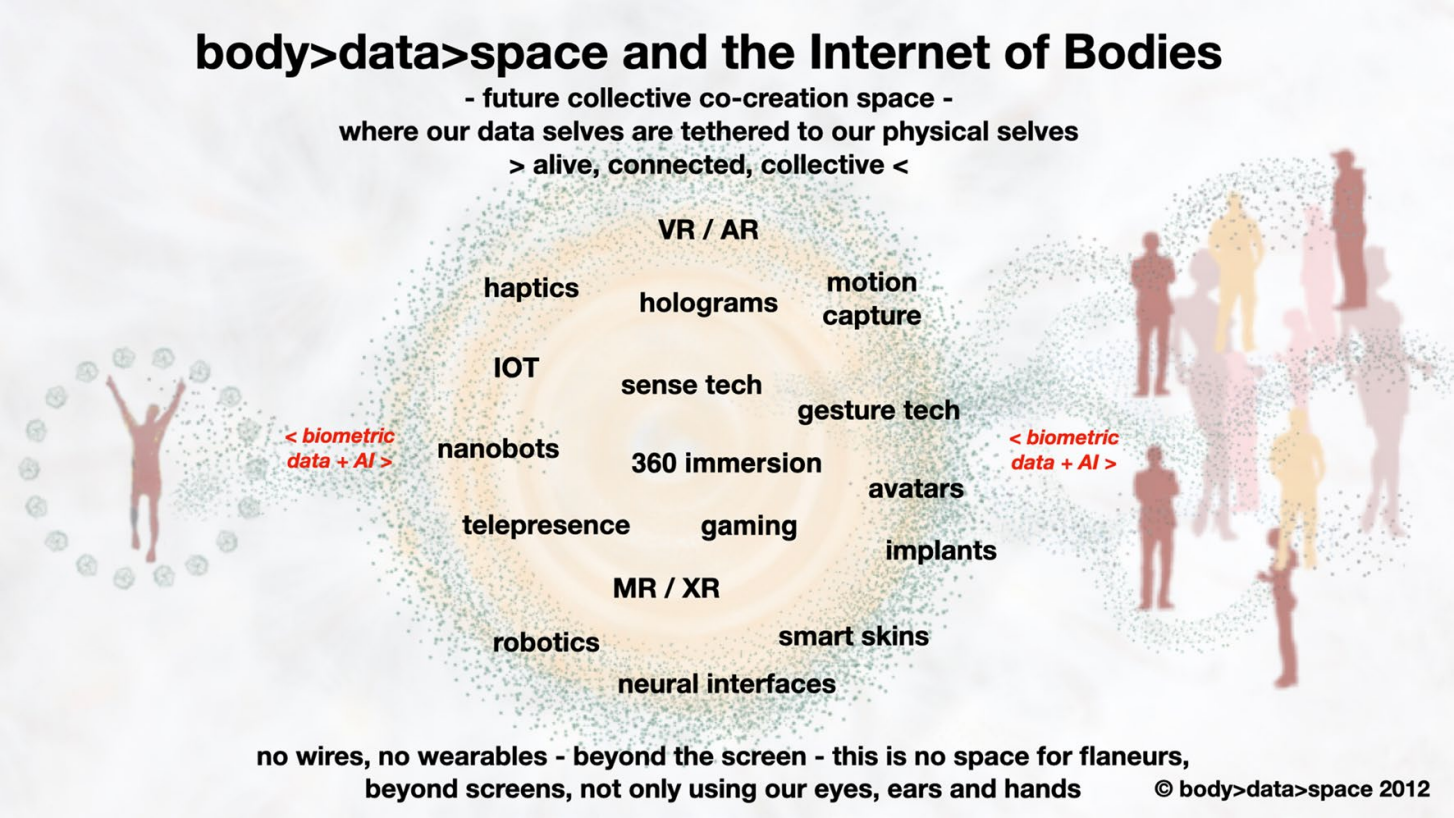
The Internet of Bodies—the future of collective co-creation spaces where our data selves are tethered to our physical selves—body > data > space 2012

Figure 10 shares my visualisation of experiential-based, collective co-creation spaces for the future, a space for the simultaneous and real-time convergence of the body and the technologies of the body, for collective co-creation. This is not a space for flaneurs; in order to ‘experience’, one must participate. This space exists beyond the screen; it hangs between two (or more) physical and two (or more) virtual spaces, created by the intersection of multiple locations.

This space is beyond the exclusivity of the triage of constant computerised action—through our eyes, ears and fingers. It challenges the assumption of this triage as the imperative created by the designers of the tools of the digital revolution. It radically advocates the use of the whole of our bodies in data immersion. The collective participation in these co-creation scenarios allows a full gamut of actual interaction and being. It enables self-presence within collective presence, and therefore a reinstatement of interconnected human synergies, a reciprocal social presence, recognisably missing from today’s popular platforms.

Here, in this collaborative blended space of body and technological convergence, we start to evolve our ability to flow with ease through a life of virtual physical blended presence. This points to major transformations, not only to our bodies but also to our understanding of ourselves, our identities and our relationship to the ‘other’. It points to a future in which we inter-connect ourselves to others through a networked “multi-self,” enabled by hyper-sensory self and a deeper tele-intuitive understanding of the virtual self.

### Collective Reality—experience togetherness

One key learning across our years of work was that we needed to accept the user as our co-creator; i.e. the public participant is ultimately part of the creation itself. As makers of public participatory installations, we are, in a sense, in a continual structured improvisation with our users, and we need to constantly be aware throughout our making processes and our iterations of the flow between the output and its extension/s created by their participation.

In 2016, we created a new participatory work called Collective Reality-Experience Togetherness (2016). This work was a slight nudge at the VR sector with its obsessive trials of groups of people, all in the same physical space, yet all wearing virtual reality headsets. A solid box covering your head, eyes and ears, disabling three of your key senses to the physical world and disallowing physical collective collaboration, in my view, the ultimate disembodiment.

Collective Reality was a large-scale yet intimate installation which, using motion tracking of group activities within the space, created increasingly active visuals and sounds. The more energy and initiative you input, the more you moved together, danced together, hugged and played together—engaging with others with similar energies, friends or strangers—the more the dynamic audio visuals generated themselves into the immersion space in response.

**Fig. 11:**
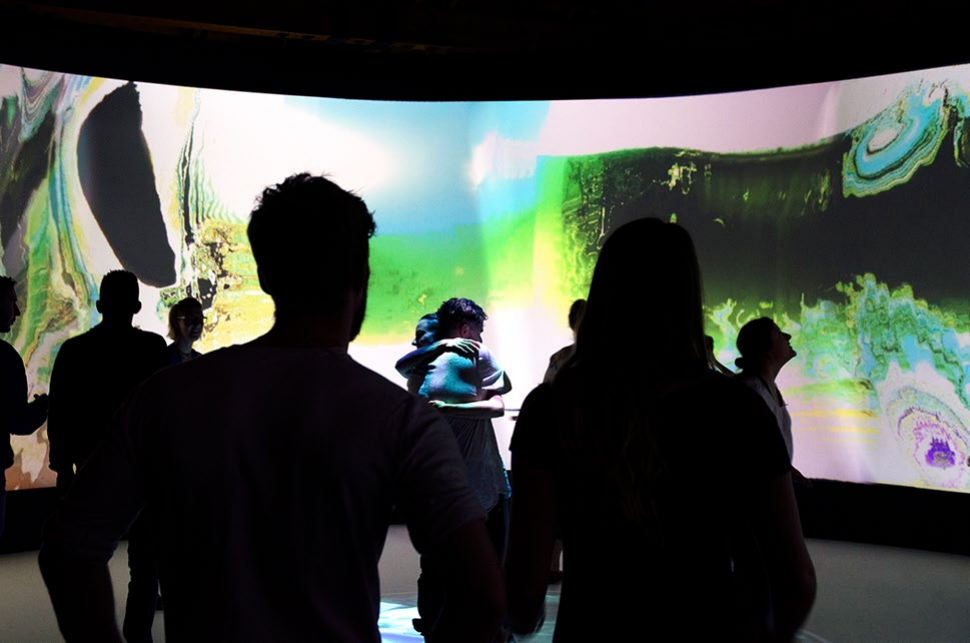
Collective Reality—Nesta FutureFest/Society for Arts and Technology (SAT) Montreal—body > data > space commission 2016

This installation came ‘alive’ when we created a physical ‘togetherness’. As aware of us as we were of it, this full-body digitally led immersion environment invited the participants to move and perform as a group. It aimed to foresee and thereby test, 10 years into the future, a real-time connected collective reality (Fig. 11).

## Extensions and convergences

This living, breathing virtual physical environment toured to the Society of Arts and Technology (SAT) Dome in Montréal to present as part of the 2017 IX Immersion Experience. The SAT Dome is a 210° dome enabling, through multiple projectors and speakers embedded in its walls, 360° immersion in sound and visuals for up to 200 people, the perfect environment for group-based physical/digital experiences (2017). Running workshops there for VR/AR software programmers (mainly male participants) we worked on helping them to understand the embodied feeling of full spatial/physical engagement within interactive generative space, shifting their understanding of their own coding through what their own physicality was yearning for in this space.

### Gesture tech and AR

The ‘Internet of Bodies’ has been extended through the use of gesture tech, using multiple biometric data feeds from the body, non-verbal signifiers and communicators which display, through a variety of actions, our feelings, our emotions and our intent.

Gesturing has been extended hugely in the gaming sector, where it has been explored in depth by games researchers and creators. For example, ‘Breath of the Wild’ (Grey 2017), which is one of the Zelda games series, depends on an important skill well recognised in Japanese culture and society—the ability to ‘read the air’. This exploits how we understand body language, facial expressions and subtle hints, used to convey information to us, more than we realise. This inferred embodied knowledge is in debate in many sectors today as being as, if not more, important than the cognitive—the thinking brain—in our communication scenarios. It queries the long-term and sci-fi-influenced debate that has been dominant from some transhumanist advocates, prioritising the brain–computer interface as the imperative, with the deletion of the physical body as an annoying encumbrance. In a way we are becoming, through digital hyper sensory awareness, more conscious of our embodied cognition, how we see, listen, feel and understand the world through our bodies (Gill 2016).

Yoichi Ochiai is a renowned young Japanese scientist and an artist working on 3D acoustic manipulation to actually project images into mid-air and to physically manipulate them (Ochiai and Hoshi 2014). The use of ultrasound waves to recreate images at a distance is also offered by Neonode in Stockholm who create tools for remote sensing solutions (2001).

Most interesting of the rapid start-ups taking place in the creative industries today is Emerge (2015), a young Los Angeles startup. They have created a non-wearable in the form of a hardware tablet which enables a tactile two-way remote digital intimacy using gesture and vibrations. With a M1 tablet in place at both ends of the transmission, one can reach out and ‘touch’ your friend, family member, partner, receiving back a visual of their hand and ‘feeling’ their touch in return. This is hand and gesture led and the most appropriate product I have seen in 2020, our year of social distancing.

### Cyborgs

Cyborg culture is also moving forward at a pace with most countries having four or five cyborgs who have reached out into media status. Manel Munoz is the weather man as such, fascinated and affected by cyclones and anticyclones, his back of the head implant sent vibrations to different sides of his head linked to weather changes around him.

Neil Harbisson from Northern Ireland calls himself a trans-species rather than a cyborg, because his implant is permanently fused into the crown of his head. He is the first trans-species/cyborg to have his passport photo accepted as he exists with his fixed antenna. Neil has, from birth, an eye condition called greyscale, which means he only sees the world in grey and white. He uses his antennae camera to detect colour, and it sends a vibration with a different frequency for each colour viewed. He is learning what colours are within his viewpoint at any given time through the vibrations in his head, a synaesthetic method of transference of one sense for another. Moon Ribas, a Spanish choreographer and a dancer, had two implants placed into the top of her feet, set to sense seismic activity as it occurs worldwide. When a small earthquake occurs somewhere, she received small vibrations; a bigger eruption gives her body a more intense vibration. She dances as she receives and reacts to these transferred data. She feels a need to be closer to our earth, a part of nature (Harbisson et al. 2018).

These cyborgs and many others are very clear about their esoteric, seemingly eccentric choices, to be part of the world in its present day condition—one of people, technology and nature having to live together. They advocate for a deeper understanding of the need to enable the new to support the existing eco-system.

### Medical, non medical and sub-dermal implants

Medical implants, embedded into the body or subdermally (nearer the surface), have rapidly advanced in the last 30 years with extensive use of cardiac pacemakers, hip implants, implantable drug pumps and cochlear implants helping partial deaf people to hear.

Deep body and subdermal implants can be personalised to your own needs. They can be set to transmit chosen aspects of your body data outwards, but they also can receive and control data in return. There are about 200 medical implants in use today. Some are complex, like deep brain stimulation for motor neurone disease, and others we are more familiar with, for example, pacemakers. Most medical implants are not digitally linked to the outside world at present, but this is in rapid evolution.

Kevin Warwick, a pioneer in this area, has interconnected himself and his partner with implants for joint use of their personal and home computer systems through their BrainGate (Warwick 2008) implant, an interface between the nervous system and the technology. They are connected bodies. He works onwards with his experiments to feel the shape of distant objects and heat through fingertip implants.

‘Smart’ implants into the brain for deep brain stimulation are in use and in rapid advancement. The ethics of these developments is under constant debate in 2020 and will be onwards, as is proved by the mass coverage of the Neuralink, Elon Musk’s innovation which connects to the brain via wires, with the initial aim to cure human diseases such as dementia, depression and insomnia and onwards plans for potential treatment of paraplegia (Musk 2016).

“Musk described Neuralink’s chip, which is roughly 23 mm (0.9 in.) in diameter, as ‘a Fitbit in your skull with tiny wires.’” says an article in NBC News (2020).

The hacking of our brain implants for memories and thoughts, or other implants for private medical information or to effect delivery is a natural current concern for the public, as security extensions to backend encryption are constantly being explored and rehacked.

I have an ImpliCaspian (Schaffgotsch 2019) implant in the back of my hand between my thumb and first finger, which links to an app and allows medical officials to see my vital medical information in an emergency. It is a bioglass capsule with a 2-KB memory working through NFC (near-field communication). It stores at the back end information in forms and additional attached pdfs holding  further details. The implant data is protected by homomorphic encryption, which preserves the encryption structure, without revealing the data, whilst it is being processed through various outsourced storage/s, a highly necessary privacy-preserving function.

There is a burgeoning interest in these non-medical implants, especially in the younger generations—personalised for our own needs and able to replace several day-to-day requirements, such as keyfobs, travel and cash cards, or enabling us to open our phones, laptops and homes with gestural swipes. I have curated and presented as part of Implant parties where live microchip implants are led by a specialist (Boddington 2019).

Also, in the corporate sector there is a trend towards inserted micro-chips for smart ticketing, banking and personal medical information. In Scandinavia many workplaces have moved forward on implanting staff, with permission, to deal with clocking in, clocking out, gym access, health bar access amongst other things. Dr. Moore (2017) writes about the various problems, including workers’ rights issues linked to workplace health initiatives involving sensory tracking devices, to electronic performance monitoring and surveillance in factories.

## The Internet of Bodies—the body is the interface

The Internet of Bodies relies on the public take up of non-medical subdermal implants as improvements for us as humans, to create and activate a hyper-enhanced human self. Exploring these topical concerns regarding the harvesting of our biometrics—our behavioural and emotional patterning as we move through the world as living beings—we can envision a unique digital self, owned by ourselves. It is our inherent need for collaboration and connection that is defining these shifts towards the inter-connected, hyper-enhanced self. These potential integrations and extensions to our physical selves point to a hugely transformative alteration of our understanding of self and other, of our identity and our agency in this world.

### Personal data ownership

I am a personal body data ownership advocate. I do believe my heartbeat is mine, my breath is mine, that my location is my private information, as is my expenditure, my health and social networks. I used to carry an organ donor card in my purse to clarify this, no longer needed from May 2020 as Britain moved to an opt out, rather than an opt in, system.

Our relationship to our personal body data in 2020—who owns it, who has the rights to use it and who controls that usage—has finally reached the forefront of the data ethics debate, and the rhetoric around data privacy and ownership has significantly shifted. With rising awareness of the attachment of our personal body data to our identity emerging from the fast release of, and intensive media debate about the COVID-19 contact tracing apps, the non-transparent issues surrounding personal data usage have reached the public in this pandemic year. Should these apps function based on centralised or decentralised systems, should they use Bluetooth and GPS to monitor and track our movements and contacts? These are issues being debated by a public suspicious and, in some cases, not willing to sign up to these apps built seemingly for common good. Medical and location data are regarded as the most private of all data by the wider public, and we have become far more enlightened to exactly how much these data do give away about us, our lives, where we go, how we behave and who we encounter—our attentions and our intentions.

The surveillance of our behaviour has led to an extensive and growing marketplace in personal body data, an economy based on ‘predicting’ the future scenarios of our lives to produce and sell us, through interpretation, what is understood as our needs and wants. These predictions are linked to machine learning led interpretations of huge collections of data garnered from our micro expressions and patterns of movement, including eye tracking, facial recognition, touch, motion, gesture, gait and location, amongst others. These data are gathered in our daily lives by the multi-surveillance camera systems surrounding us in public spaces such as airports and shopping centres, by the sensors in our homes, in and in our workplaces, and by our wearables and immersion tools such as virtual and augmented reality experiences.

This is clarified by studies of people moving within 360° VR, results showing that in 5 min one can identify an individual from their motion data, without any other identifiers attached to that data (Bailenson 2018). Much of these bio-data are gathered from us “invisibly”, with private companies harvesting our information and selling it to others as a valuable asset of our engagement patterns, often without our knowledge, making and retaining for themselves sizable profits from the personal body data of each individual in the world.

This brings to the forefront further questions about our identity and our self-hood and how we will estimate our responsibility in/to the world, as the actuality is that our data and therefore ‘ourselves’ are seemingly ‘owned’ by others. The concern is that this scenario will lead to a separation of ourselves from our self-responsibility, as our personal body data starts acting independently from ‘ourself’, through its harvested pathway of being bought, sold on and used in numerous different ways.

Although we mostly tend to believe in the use of the gathering of big data to analyse and create solutions for social good, no longer is everyone prepared to hand over their personal data without a better and more precise understanding of its use beyond ourselves, in particular to government and/or privately owned health or other sector servers.

### How do we get a win–win situation?

When presenting to corporate audiences, I highlight that we need to work together to ensure a win–win situation. Companies, both large and small, spend much time and effort thinking up new methodologies to make and retain profit from using their customers personal data. It is clear that, often unknowingly, we all have our data (mis)used and this includes the data of the company directors themselves, and their families. These digital products and services are sold to us as complementary to our needs, to enable us to be enhanced through interactions across the world, across time and space.

They need to fulfil this offer honestly, not to be designed with an intent to make high return revenue streams from the sale of our personal data, gathered at the back end of these innovations. This revenue, worth tens of thousands of pounds a year per person, never reaches us as individuals, and is significantly responsible for the rising divide between rich and poor, both in material wealth and digital access.

Yet as technology moves inside our bodies, this debate needs serious consideration within a wider public context, as there are many questions in regards to this seemingly unstoppable collection and exploitation of our personal body data:

1. What are the positive and negative implications of connected implants embedded into humans?
2. Are such technologies only to be seen as invasive and dehumanising, or are they a logical development of medical implants and data led bio-interactive wearables?
3. What are the potentials of evolving trusted encryption methodologies in relationship to our personal data rights, to secure our protection from hacking and from bio-data replicas and deep fakes?
4. How will we, with our future implants bio-connecting us, work and share into a collective collaborative space?
5. How can we, through this process, support inclusivity and eliminate biases, and extend and embrace the deep value of our diversities?
6. As artificial intelligence merges with human intelligence, how do we create a sound moral and social framework for this collaboration?
7. What universal compliance could be developed amongst nations to support and eliminate the trust and privacy issues?

One can view many positive opportunities linked to our data becoming implanted into our living bodies, with one clear proviso—for this to succeed and not result in the dystopian sci-fi-led view of human control through embedded technologies, we do need to secure personal data sovereignty.

**Fig. 12:**
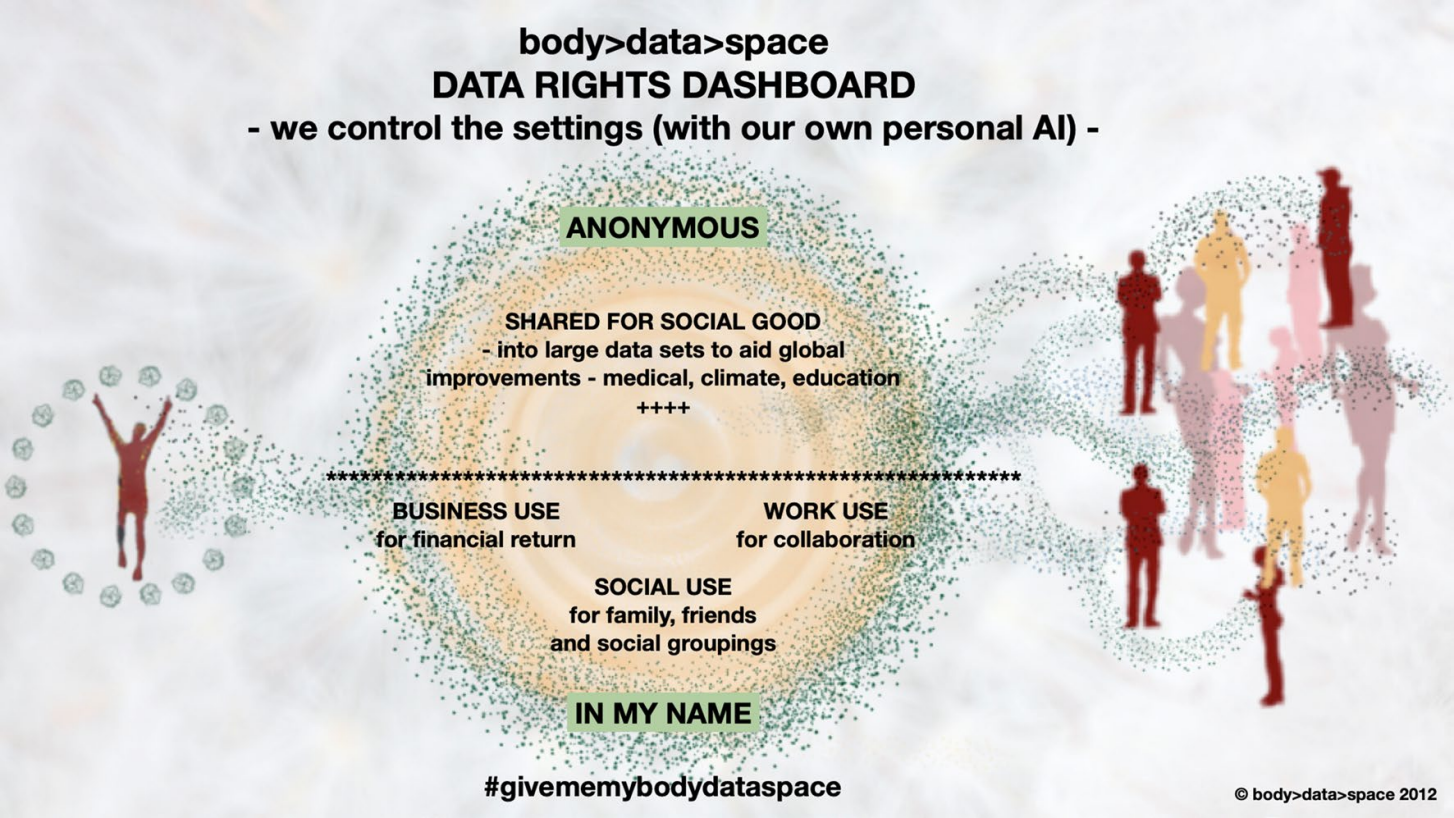
Personal Data Dashboard—body > data > space 2012

Figure 12—personal AI data dashboard is a simple diagram to show, by example, how we could each access and control our own data, using our ‘self’ to make our own choices. as unique beings. We would have the choice to share our data anonymously into crowdsourced big data pools, to benefit the community. The COVID-19 virus again is a prime example of this, as the exchange of mass data has enabled a rapid sharing of knowledge, to create crowdsourced wisdom for the world, resulting in vaccinations being developed by scientists and approved for safe use into the population by governments at a rate previously unimaginable. This type of collaborative and collective action can enable us to tackle other huge global issues, such as climate change and regional development needs.

Figure 12 also illustrates that, most imperative I believe, we should also have the choice to withhold or charge for our data.

### Data ethics and future needs

Data Unions and personal data stores (2020) are evolving, platforms created to enable companies to pay users for their data, so that they can still develop better products and predict our needs, yet reward us for the behavioural and emotional data outputs we (choose) to supply—a fairer trade-off than the present scenario. I put forward the following set of baseline actions required by company culture:

- any company developing any body interfacing technology must place a body expert at the core of its development team, from conception stage onwards, be it a neurologist, a biologist, a dancer or a sports person, depending on the emphasis of their product or service
- every company, whatever size, should have a transparent data ethics governance declaration around the use and protection of customers’ private and personal data
- all companies need to be transparent about this upfront on their websites (and not at the back end, with multiple pages of Terms and Conditions)
- companies should actively involve themselves in the data union solution debate and take urgent action to find positive solutions.

Younger generations today are already showing an understanding of the requirements laid out above and are beginning, both for sustainability and environmental concerns as well as data privacy issues, to research deeper into the products they buy, the companies they work for and how their personal data is being used. I believe the above points will become clear USPs (unique selling points) for consumers searching across a wide range of products and services, aiding them to make decisions on which company they choose to purchase from (Ustinova 2020).

## Conclusions—my AI data body belong to me, and is tethered to my physical body as part of my collective collaborative future

I envision a future where my data body is tethered to my physical body, working in alliance with my own companion AI, connecting me, through my implants and sensors, to collective co-operations and conversations. This can all be controlled from my personal data dashboard (see Fig. 12) and, through this process, I have sealed a secure bond between my physical self and my multiple ‘other’ data selves, across space and time.

It is the convergence of embeddable technologies with personal data ownership that I advocate as the way forward, augmented in the future by our own personalised AI, taking our identity as the key protective, above and beyond the use of any company or government without our permission.

Embedded technologies will allow us to hold our own data within our own bodies—not on a server or a metaverse owned by profiteers, not taken from a wearable that sells our body data with no return to us. Implants can attach our data to us, ensuring it is part of us. They enable our data to move with us, solving many problems for the displaced amongst us. They will give us the ability to be more deeply interconnected, traversing distance and time, to work, play and share knowledge, wisdom and creativity with other humans globally, and from the depths of our beings.

As this vision of the bio-connected hyper-embodied human starts to become reality it potentially can, with open debate, make positive steps towards the human self we would wish to be, by evolving this through the ethical use of the technologies we have created to enhance and augment ourselves, our senses, our perceptions and our awareness.

Professor Ascott (1984) wrote about computer-mediated networks offering ‘the possibility of a kind of planetary conviviality that no other means of communication has been able to achieve. One reason may be that networking puts you, in a sense, out of body, linking your mind to a kind of seamless sea’—which he refers to as a Jungian ‘collective unconscious’.

My proposition embraces and extends this concept, to include a deeper integration between our ‘out of body’ and our inner sensory experiences, one that interlinks our body and our mind, that regards the non-verbal as of equal importance, as proved by the emergence of the financially valuable behavioural economy.

This can network us into the ‘seamless sea’, alongside the rest of humanity as collective virtual and physical embodiments, as an Internet of Bodies. And here we need to prioritise intimacy, a key human need as proved by the social distancing effects on humanity during the pandemic. Through the blending of the virtual and the physical body, we can enable collective embodiment to work holistically with collective intelligence, towards positive sensory enhancement.

Here, I can exist physically as my physical self, in collaboration with my tele-present selves, my avatar selves and my holographic selves, transmitting my presence and expanding my identity, across time and space—now blended into a hypersensory self.

The multiple-layered conversation that would exist between our connected and collective embodiment, alongside our collective intelligence, could help restore a sense of innate responsibility, to ourselves and to our data, to others as well as to ourselves. This reprioritises the rising need for privacy, linked to our identity and therefore to our dignity—a baseline human right.

It is clear that today that finally the body is the interface; it is the personal ownership of this interface that we need to reclaim.
